# Single Endemic Genotype of Measles Virus Continuously Circulating in China for at Least 16 Years

**DOI:** 10.1371/journal.pone.0034401

**Published:** 2012-04-20

**Authors:** Yan Zhang, Songtao Xu, Huiling Wang, Zhen Zhu, Yixin Ji, Chunyu Liu, Xiaojie Zhang, Liwei Sun, Jianhui Zhou, Peishan Lu, Ying Hu, Daxing Feng, Zhenying Zhang, Changyin Wang, Xueqiang Fang, Huanying Zheng, Leng Liu, Xiaodong Sun, Wei Tang, Yan Wang, Yan Liu, Hui Gao, Hong Tian, Jiangtao Ma, Suyi Gu, Shuang Wang, Yan Feng, Fang Bo, Jianfeng Liu, Yuan Si, Shujie Zhou, Yuyan Ma, Shengwei Wu, Shunde Zhou, Fangcai Li, Zhengrong Ding, Zhaohui Yang, Paul A. Rota, David Featherstone, Youngmee Jee, William J. Bellini, Wenbo Xu

**Affiliations:** 1 WHO WPRO Regional Reference Measles Lab, National Institute for Viral Disease Control and Prevention, China Center for Disease Control and Prevention, Beijing, China; 2 Institute of Pathogen Biology, Chinese Academy of Medical Science & Peking Union Medical College, Beijing, China; 3 Changchun Children's Hospital, Changchun, Jilin, China; 4 Jilin Provincial Center for Disease Control and Prevention, Changchun, Jilin, China; 5 Jiangsu Provincial Center for Disease Control and Prevention, Nanjing, Jiangsu, China; 6 Henan Provincial Center for Disease Control and Prevention, Zhengzhou, Henan, China; 7 Shandong Provincial Center for Disease Control and Prevention, Jinan, Shandong, China; 8 Guangdong Provincial Center for Disease Control and Prevention, Guangzhou, Guangdong, China; 9 Shanghai Provincial Center for Disease Control and Prevention, Shanghai, China; 10 Liaoning Provincial Center for Disease Control and Prevention, Shenyang, Liaoning, China; 11 Hebei Provincial Center for Disease Control and Prevention, Baoding, Hebei, China; 12 Shanxi Provincial Center for Disease Control and Prevention, Taiyuan, Shanxi, China; 13 Tianjin Provincial Center for Disease Control and Prevention, Tianjin, China; 14 Ningxia Provincial Center for Disease Control and Prevention, Yinchuan, Ningxia, China; 15 Innermongolia Provincial Center for Disease Control and Prevention, Huhehaote, Innermongolia, China; 16 Zhejiang Provincial Center for Disease Control and Prevention, Hangzhou, Zhejiang, China; 17 Heilongjiang Provincial Center for Disease Control and Prevention, Haerbin, Heilongjiang, China; 18 Gansu Provincial Center for Disease Control and Prevention, Lanzhou, Gansu, China; 19 Shannxi Provincial Center for Disease Control and Prevention, Xi'an, Shannxi, China; 20 Anhui Provincial Center for Disease Control and Prevention, Hefei, Anhui, China; 21 Guangxi Provincial Center for Disease Control and Prevention, Nanning, Guangxi, China; 22 Guizhou Provincial Center for Disease Control and Prevention, Guiyang, Guizhou, China; 23 Jiangxi Provincial Center for Disease Control and Prevention, Nanchang, Jiangxi, China; 24 Hunan Provincial Center for Disease Control and Prevention, Changsha, Hunan, China; 25 Yunnan Provincial Center for Disease Control and Prevention, Kunming, Yunnan, China; 26 Hubei Provincial Center for Disease Control and Prevention, Wuhan, Hubei, China; 27 Division of Viral Diseases, Centers for Disease Control and Prevention, Atlanta, Georgia, United States of America; 28 Immunization, Vaccines and Biologicals, World Health Organization, Geneva, Switzerland; 29 Expanded Programme on Immunization, Western Pacific Regional Office, World Health Organization, Manila, Philippines; British Columbia Centre for Excellence in HIV/AIDS, Canada

## Abstract

The incidence of measles in China from 1991 to 2008 was reviewed, and the nucleotide sequences from 1507 measles viruses (MeV) isolated during 1993 to 2008 were phylogenetically analyzed. The results showed that measles epidemics peaked approximately every 3 to 5 years with the range of measles cases detected between 56,850 and 140,048 per year. The Chinese MeV strains represented three genotypes; 1501 H1, 1 H2 and 5 A. Genotype H1 was the predominant genotype throughout China continuously circulating for at least 16 years. Genotype H1 sequences could be divided into two distinct clusters, H1a and H1b. A 4.2% average nucleotide divergence was found between the H1a and H1b clusters, and the nucleotide sequence and predicted amino acid homologies of H1a viruses were 92.3%–100% and 84.7%–100%, H1b were 97.1%–100% and 95.3%–100%, respectively. Viruses from both clusters were distributed throughout China with no apparent geographic restriction and multiple co-circulating lineages were present in many provinces. Cluster H1a and H1b viruses were co-circulating during 1993 to 2005, while no H1b viruses were detected after 2005 and the transmission of that cluster has presumably been interrupted. Analysis of the nucleotide and predicted amino acid changes in the N proteins of H1a and H1b viruses showed no evidence of selective pressure. This study investigated the genotype and cluster distribution of MeV in China over a 16-year period to establish a genetic baseline before MeV elimination in Western Pacific Region (WPR). Continuous and extensive MeV surveillance and the ability to quickly identify imported cases of measles will become more critical as measles elimination goals are achieved in China in the near future. This is the first report that a single endemic genotype of measles virus has been found to be continuously circulating in one country for at least 16 years.

## Introduction

Measles continues to be a leading cause of childhood morbidity and mortality in developing countries and an outbreak threat in the majority of countries, despite the availability of an effective vaccine for over 40 years [1]. Globally, major reductions have been achieved in measles mortality, from an estimated 733,000 deaths in 2000 to 164,000 in 2008 [2].

Measles virus (MeV) is a negative-sense, single-stranded RNA virus in the genus *Morbillivirus* within the family *Paramyxoviridae*. MeV is serologically monotypic, but genetic variability exists among wild type strains [3]. The World Health Organization (WHO) currently recognizes 23 genotypes and one provisional genotype of MeV [4], [5] based on sequence analysis of the 450 nucleotides (nt) that code for the 3′ region of the nucleoprotein (N) gene (N450) and 1854 nt that code for the entire hemagglutin (H) gene [6], [7], [8], [9].

**Figure 1:**
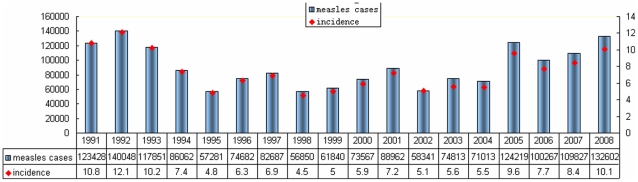
Number of measles cases and incidence from 1991 to 2008. Source: National Notifiable Diseases Reporting System (NNDRS). Blue bars indicate the reported measles cases and red solid diamonds indicate the incidence (/100,000 ) of each year. X-axis denotes year, left-hand y-axis denotes reported number of cases and right-hand y-axis denotes the incidence per 100,000.

Molecular epidemiological studies of MeV have made significant contributions to measles control efforts, by providing a means to confirm the sources of virus or suggest a source for unknown-source cases as well as to establish links, trace the transmission pathway, allow discrimination of imported from indigenous measles cases, or classify suspected cases as vaccine reactions [10], [11], [12]. Molecular surveillance is most beneficial when there is continuous high quality surveillance and it is possible to observe the change in viral genotypes over time in a particular geographical region. Since this information can help to document the interruption of transmission of MeV, molecular surveillance can provide an important method to assess the effectiveness of vaccination programmes.

Measles is a vaccine-preventable disease, but is still a major killer of children worldwide. Measles caused many outbreaks in China before the 1965 introduction of liquid measles vaccine (MV) administered as 1 dose to infants aged >8 months. The National Expanded Program on Immunization (EPI) was established in 1978 to allow routine immunization with MV to cover the entire country [13], [14]. During 1986–2004, a 2-dose schedule using lyophilized MV at 8 months and at 7 years of age was introduced [6]. The targeted population of the routine immunization has been adjusted to 8 months and 24 months of 2 doses since 2005. When the Universal Childhood Immunization goals were attained, measles morbidity and mortality decreased markedly [13], [15].

The WHO Regional Committee of the Western Pacific Region (WPR) formally declared a measles elimination goal in 2005 and established a target date of 2012 for regional measles elimination [16]. In China, MeV surveillance has been established since 1993, and a serological surveillance laboratory network was established in 2001 to provide serological confirmation of measles infection by IgM testing and to support measles surveillance [14], [15]. To reach the goal of measles elimination by 2012 in China, the Ministry of Health (MOH) proposed a comprehensive vaccination and surveillance programme in 2006 (Measles Elimination Action Plan from 2006–2012 issued by MOH). The "Measles elimination action plan" defines maintenance of a high routine coverage of the first measles containing vaccine (MCV) dose at 8 months of age and the second MCV dose at 18–24 months combined with supplemental immunization activities among individuals aged 8 months to 14 years [14]. We have been studying the molecular epidemiology of MeV circulating in China since the 1990s [13], [15], [17], [18]. In this study, we reviewed the incidence of measles cases in China during 1991 to 2008 and analyzed the sequences of a collection of 1507 measles isolates collected between 1993 and 2008. Viruses of the single endemic genotype, H1, were found in different geographical areas of China over the period of at least 16 years and a major shift from H1b to H1a was documented. This study investigated the genotype and cluster distribution of MeV in China over a 16-year period to establish an important genetic baseline before MeV elimination in the Western Pacific Region (WPR).

## Materials and Methods

### Ethics Statement

This study did not involve human participants or human experimentation; the only human materials used were throat swab or urine samples collected from patients with acute, febrile maculopapular rash at the instigation of the Ministry of Health P. R. of China for public health purposes, and written informed consent for the use of their clinical samples was obtained from all patients involved in this study. This study was approved by the second session of the Ethics Review Committee of the Chinese Center for Disease Control and Prevention.

### Epidemiologic data

Reported numbers and incidence of measles cases and deaths in this report were obtained from the National Notifiable Diseases Reporting System of China CDC (NNDRS). Population denominators for calculation of incidence and mortality rates were determined on the basis of data reported by the National Bureau of Statistics. Epidemiologic data were analyzed by using Microsoft Excel.

### Specimen collection and Virus isolation

Throat swab or urine samples were collected from patients with acute, febrile maculopapular rash. All clinical samples were collected within five days of rash onset and transported to the laboratory in accordance with standard protocols [19]. Isolation of MeV was performed using the B95a [20] or Vero/hSLAM [21]cell line and the infected cells were harvested when the cytopathic effect (CPE) was visible over at least 75–90% of the cell layer [19].

### RNA Extraction and RT-PCR

RNA was extracted from 250 μl of infected cell lysate using a Trizol reagent, following the manufacturer's instructions. For all virus isolates, RT-PCR amplification was performed using previously described primers to amplify a 600 bp fragment in the N gene which included the 450 bp fragment recommended for genotyping [10]. PCR products were purified using the QIAquick Gel Extraction kit (QIAGEN).

### Phylogenetic analysis

Sequences of the PCR products were derived by automated sequencing and the BigDye terminator v3.0 chemistry according to the manufacturer's protocol in both sense and antisense strands by an automated ABI PRISM™ 3100 DNA Sequencer (Perkin Elmer). Sequence proof reading and editing was conducted with Sequencer™ (Gene Codes Corporation). Sequence data were analyzed using version 7.0 of Bioedit and phylogenetic analyses were performed with the evolution model of Maximum Composite Likelihood using Mega 4. The robustness of the groupings was assessed using bootstrap re-sampling of 500 replicates and the trees were visualized with Mega programs.

### Parameter estimates and statistical analysis of the amino acid sites under selection pattern

Synonymous and non-synonymous substitutions: to determine whether the detected mutations in wild type MeV were the consequence of selection pressure, the frequencies of synonymous (dS) or non-synonymous (dN) was calculated, which is termed as ω, the (average) dN/dS rate ratio. An ω<1 suggests purifying (negative) selection, whereas an ω>1 is indicative of positive selection because that the rate of fixation is higher than the background rate of mutation, which defies neutrality (ω = 1)[22]. The Nei-Gojobori (p-distance) method (MEGA Versions 4), was employed to calculate ω ratios and to identify amino acid sites as conserved, neutral or positively selected [22].

### Nucleotide sequence accession numbers

The nucleotide sequences of 135 viruses, representative of the 333 Chinese MeV of 2008 that were isolated in this study, were deposited in the GenBank database under accession numbers JN380206-JN380341. An additional 692 viruses used in this study were previously deposited in GenBank (accession numbers HAF045191-HAF045218, DQ356683- DQ356873, EU557194- EU 557238, FJ602549- FJ 602674, GU237175- GU 237481).

## Results

### Measles incidence and mortality rates in China

The system of reporting infectious diseases, including measles, to the National Notifiable Disease Reporting System (NNDRS) had been established in the 1950's in China. The cases of disease were reported by posting a card monthly from the hospitals to the national level (sequentially, from county Centers for Disease Control (CDC), to prefecture CDC and provincial CDC, to the national level). Since 2004, the NNDRS has been updated to allow hospitals or local CDCs to directly report all suspected measles cases immediately to the national level through a web-based, real-time reporting system.

With the attainment of childhood immunization goals of 2 doses of measles vaccine since 1986, the measles morbidity and mortality have decreased markedly. Subsequently, measles epidemics occurred about every 3–5 years, with the incidence fluctuating around 8/100,000. The average annual reported incidence in 1994–2004 was below 7.5/100,000 (average, 5.84/100,000; range, 4.5[1998]–7.4[1994] /100,000). But measles cases resurged in 2005–2008 and the average annual reported incidence (average, 8.95/100,000; range, 7.7[2006]–10.1[2008]/100,000) represented a 53.3% increase compared with 1994–2004 (Fig. 1.). However, the average annual reported number of measles deaths in 2005–2008 (average 65; range 35–103), decreased 55.5% compared with 1994–2004 (average 146; range 23–250).

### Determination of MeV genotypes in China

**Figure 2:**
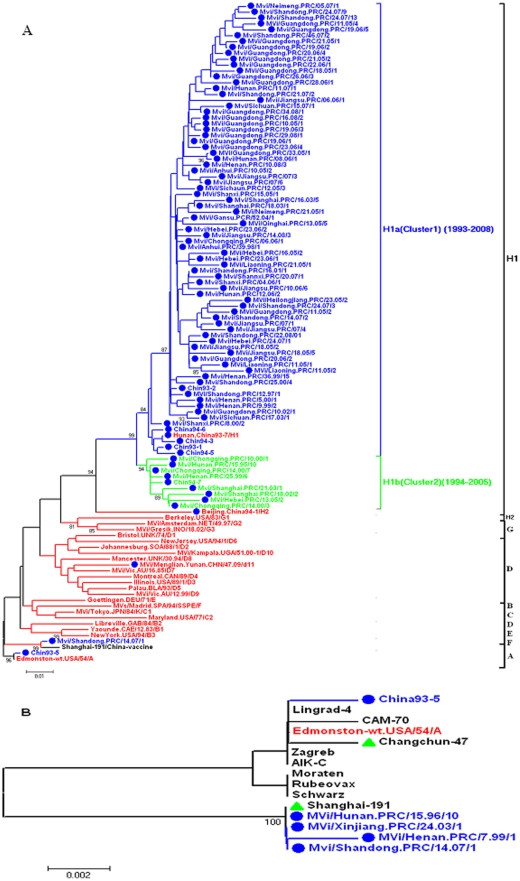
Phylogenetic analysis of sequences of representative Chinese measles viruses, compared to the WHO reference sequences. These trees are based on the WHO standard sequence window within the N gene. Panel A. Phylogenetic tree of 87 representative measles isolates from China during 1993–2008 compared to the WHO reference sequences for each genotype. Sequences from Chinese viruses of H1a cluster from 1993–2008 are indicated by blue and sequences from Chinese viruses of H1b cluster from 1994–2005 are indicated by fluorescence green, and WHO reference strains are indicated by red. All isolates from China, including 3 WHO reference strains(Hunan.China93-7/H1, Beijing.China94-1/H2, MVi/Menglian.Yunnan.CHN/47.09/d11), are indicated by solid rounded dots. Panel B. Phylogenetic tree of 5 measles vaccine viruses from China compared to the A genotype strains of Edmonston wild type and other vaccine strains used worldwide. Sequences from viruses isolated in China are indicated by blue, and A genotype wild type Edmonston strain is indicated by red. Two Chinese measles vaccines are indicated by green solid triangles.

A total of 1507 measles viral isolates were identified from throat swab or urine specimens obtained from 30 of 31 provinces in China (except for Tibet), between 1993 and 2008 (Table S1). The throat swab or urine specimens were collected from measles outbreak or sporadic cases. Virus isolation was conducted by provincial laboratories and isolates were transported to the National Measles Laboratory (NML) in Beijing for molecular characterization. The N450 nucleotide sequences from the 1507 measles viruses in this study were compared to the 23 WHO reference viruses which include two wild-type viruses from China representing H1 genotype (MVi/Hunan.China/93/7/H1)and H2 genotype (MVi/Beijing.China/94/1/H2). With the exception of one H2 genotype and five A genotype strains, all other 1501 strains were H1 genotype (Table S1). These genotype assignments were supported by high bootstrap scores (Phylogenetic tree not shown). The sequences of virus isolates from each outbreak and some sporadic cases were identical or very similar. A group of 87 of 1501 H1 genotype strains were selected on the basis of their genetic variability (strains ≥0.5% nt difference ) as representative viruses for the construction of the phylogenetic tree shown in Fig. 2. All viruses were named according to the WHO systematic nomenclature for measles viruses (26).

Another phylogenetic tree was constructed with all H1 sequences between 1993 and 2008, which showed that all the H1 sequences could be divided into 2 groups that we propose as H1a (with 1445 strains) and H1b (with 56 strains) clusters. Based on the WHO standard 450 nucleotide sequencing window in the N gene, a 4.2% nucleotide divergence was found between the H1a and H1b clusters, and the nucleotide sequence and predicted amino acid homologies of H1a viruses were 92.3%–100% and 84.7%–100%, H1b were 97.1%–100% and 95.3%–100%, respectively. The greatest nucleotide variation among all H1 strains was 8.6% between a H1a strain, Mvi/Guangdong.PRC/19.06/5 and a H1b strain, MVi/Hebei.PRC/13.05/2.

### Chronological and geographical distribution of Chinese MeV genotypes

Two clusters of measles viruses were found in China during three time periods (1993 to 1994, 1995 to 2005, and 2006 to 2008). Nucleotide sequences from 14 viruses were available between 1993 and 1994, those from 569 viruses were available between 1995 and 2005, and those from 920 viruses were available between 2006 and 2008. MeV isolates were obtained from most provinces since the year 2000 (Table S1).

**Figure 3:**
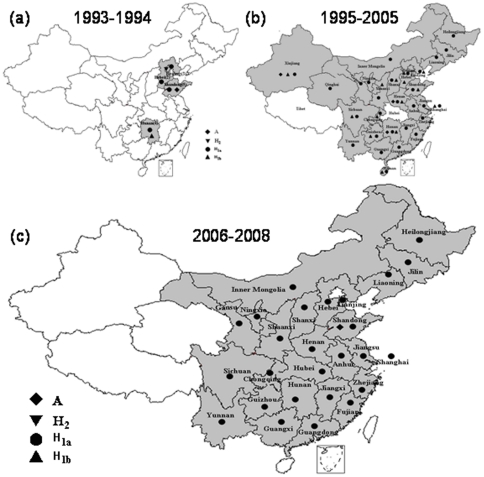
The geographical distribution of the genotypes and cluster of measles viruses isolated in China during three periods between 1993 and 2008. The provinces where the measles viruses of the indicated genotypes (clusters) were found are shown. The location within each province is not indicated. Genotype H2 viruses in Beijing may be classified as imports, and 5 genotype A viruses in four provinces are vaccine associated (see the text).

**Figure 4:**
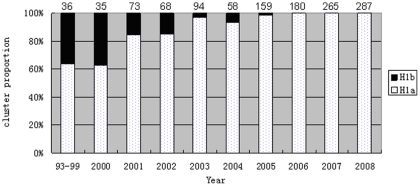
Different epidemic trend of 2 clusters of H1 genotype viruses circulating in China during 1993–2008. The number on the top of each column represents the number of the isolates in each time period.

The geographical distribution of Chinese MeV genotypes and clusters are shown in Fig. 3. Wild type measles viruses of genotypes H1 and H2 were found during 1993 to 1994 in Beijing, Shandong, Hunan, and Hebei provinces and one vaccine-like virus was detected in Shandong province in 1993. Except for four S191 vaccine-like A genotype viruses, all viruses during 1995 to 2008 were of genotype H1. During 1993 to 2008, different epidemic patterns of two clusters of H1 genotype were found. Two clusters of H1 viruses (H1a, H1b) co-circulated in 15 provinces (Beijing, Chongqing, Guizhou, Hainan, Hebei, Henan, Hunan, Shannxi, Shandong, Shanghai, Shanxi, Sichuan, Tianjin, Xinjiang, Yunnan) during 1993–2005; and only H1a cluster viruses were found during 2006–2008, in 28/31 provinces, except for Qinghai, Xinjiang and Tibet. (Fig. 4). Viruses from both clusters were distributed throughout China with no apparent geographic restriction and multiple co-circulating lineages were present in many provinces.

Five genotype A viruses were found from 1993 to 2007: Shandong in 1993 (MVi/Shandong,CHN/9353); Hunan in 1996(MVi/Hunan.PRC/15.96/10); Henan in 1996(MVi/Henan.PRC/7.99/1); Xinjiang in 2003 (MVi/Xinjiang.PRC/24.03/1) and Shandong in 2007(MVi/Shandong.PRC/14.07/1). There was one nucleotide difference between MVi/Shandong,CHN/9353 and Edmonston-wt.USA/54/A, and one nucleotide difference between MVi/Shandong,CHN/9353 and vaccine strain AIK-C and Zagreb as well. The other four genotype A strains were genetically close to the Chinese measles vaccine strain, S191, with identical or one nucleotide difference comparing with S191.

### Parameter estimates and statistical analysis of the sites under positive selection

The frequencies of synonymous (dS) and non-synonymous (dN) were calculated using the Nei-Gojobori method with MEGA Version 4.0 based on the N gene 3′ region. Eighty one H1a and nine H1b sequences shown in phylogenetic tree (Figure 2) were used for dS and dN analysis. The ratios were 0.605 and 0.195 respectively for H1a and H1b, which are all less than 1 suggesting that there is no positive selective pressure on the N 3′ end region.

## Discussion

This study summarizes the measles surveillance data and documents epidemic measles cycles occurring approximately every 3 to 5 years (1992, 1997, 2001, 2005, 2008). China experienced lower incidence for 11 years during 1994–2004 after two dose measles vaccine schedule were initiated in 1986. However, this was followed by measles resurgence during 2005–2008. Thus, the 3–5 years measles epidemic cycles were possibly associated with the fluctuation of immunity coming from the combination of natural infection and vaccine-induced immunity.

China has committed to eliminate endemic MeV transmission by 2012 [16]. It is critical to determine the genetic baseline of MeV in China before MeV elimination. This study investigated the genotype and cluster distribution of MeV in China over a 16-year period to establish the genetic baseline. Molecular epidemiology and phylogenetic analyses have become important tools in monitoring virus circulation and the progress of elimination efforts, and these baseline data will prove invaluable for classifying viruses found in China as either indigenous or imported. For example in 2009, based on the data presented here, genotype D9, D4 and d11 viruses were identified by molecular epidemiology to be non-indigenous strains and in combination with epidemiological investigations were shown to be imported from Thailand, France and Myanmar, respectively.[5], [23], [24].

Among the wild-type viruses of two genotypes detected in China between 1993 and 2008 (H2 and H1), genotypes H2 had been not found since 1993–1994. H2 genotype virus, Beijing.China94-1/H2 (later designated as the WHO reference strain) was isolated from an unvaccinated 22-year-old male from Beijing, but no epidemiologic data were available to indicate the source of infection. However, it was found to be closely genetically similar to contemporaneously circulating Vietnamese measles viruses [25], [26]. No H2 viruses were found in mainland China after 1994. The genotype A strain: MVi/Shandong,CHN/9353 should be a vaccine associated virus since no more wild type viruses were detected since recent decades. One virus, MVi/Shandong.PRC/14.07/1, was isolated from the throat swab specimen of a vaccinee, a 7 years old boy who received measles vaccine (S191) 7 days before rash onset. Although immunization histories were not available for the other three genotype A sporadic cases, the sequence information indicated that the three isolates likely came from the MeV used in the vaccination programme. Vaccine virus isolations can easily occur when outbreaks and vaccination campaigns occur simultaneously and 10% of S191 vaccinee have the side effect of fever and rash, therefore, more S191 vaccine viruses will be detected with the implementation of Supplementary Immunization Activities (SIAs) in China. Therefore, it is very important to differentiate the measles cases and vaccine associated cases rapidly to guide the control strategies.

The data presented here show that genotype H1 was the predominant circulating genotype throughout China continuously for at least 16 years. Genotype H1 has the quite great degree of intratypic variation among all genotypes, with sequences falling into two major clusters, previously designated as cluster 1 and cluster 2 [13], [15], [17] which corresponded to clusters H1a and H1b, respectively, in our study.

A 4.2% average nucleotide divergence was found between the two clusters, cluster H1a and H1b. These two clusters of viruses were co-circulating during 1993 to 2005, while cluster H1a viruses was continuously circulating after 2006. In order to rapidly increase the immunity level of population, block the spread of the virus and reduce the incidence, the province-specific SIAs had been conducted since 2004. The transmission of H1b viruses was possibly interrupted by vaccination in 2005 and that only the H1a viruses continued to circulate. With the implementation of province-specific SIAs in 2004–2009 and following synchronized nationwide SIAs throughout of China, the reported measles incidence decreased to a historically low level of 39.5–28.9 cases per million in 2009–2010. The imported D4, D9 and d11 genotype measles viruses were found, however H1 still is predominant genotype circulating in China mainland during 2009–2010(unpublished data). More and more imported non-H1 genotype measles viruses could be detected at the measles elimination stage in China in the future.

The molecular epidemiological pattern of measles viruses in China appears to be similar to mumps virus [27], human enterovirus 71 [28], and coxsackievirus A16 [29], where one predominant genotype continuously circulating in China since they were first detected in the 1990s. However, it is quite different from those of the rubella virus [30], where virological surveillance for rubella virus since 1979 showed that 5 different genotypes were cocirculating in China.

In this study, analysis of the predicted amino acid substitution in the 450 nt sequencing window of the MeV N gene showed no evidence of selective pressure in the past 16 years. Of course, the major target of neutralizing antibodies to measles virus in the H protein and it will be important to monitor genetic changes that may affect antigenic sites on this protein.

Although measles virological surveillance has been successfully implemented in China, and now included 30 of 31 provinces, systematic collection from each province and each prefecture should be encouraged to establish a baseline of complete chronological and geographical distribution of Chinese MeV for the entire country. Quickly identifying imported cases of measles will become more critical as measles elimination goals are achieved in China in the near future.

## Supporting Information

Table S1 The list of measles virus isolates in China in 1993–2008.
(DOC)
