# Supplementary material for: Single Endemic Genotype of Measles Virus Continuously Circulating in China for at Least 16 Years
Source: PLoS One. 2012 Apr 20;7(4):e34401. doi: 10.1371/journal.pone.0034401 (PMC3332093; doi:10.1371/journal.pone.0034401)
Supplement: Table S1 — The list of measles virus isolates in China in 1993–2008. (DOC) [file pone.0034401.s001.doc]

Table S1

| **Province** | **Genotype/Cluster(no. of isolates )** | **Years(no. of isolates )** |  | **Province** | **Genotype/Cluster(no. of isolates )** | **Years(no. of isolates )** |
| --- | --- | --- | --- | --- | --- | --- |
| Anhui | H1a(20) | 1998(1),2000(2),2001(3), 2002(3), 2004(2), 2005(4), 2007(3), 2008(2) |  | Jiangxi | H1a(10) | 2002(2),2006(1), 2007(5), 2008(2) |
| Beijing | H1a(2) | 1994(2) |  | Jilin | H1a(31) | 2001(5),2002(4), 2005(8), 2006(10), 2008(4) |
|  | H1b(1) | 2000(1) |  | Liaoning | H1a(50) | 2000(1),2001(5), 2002(4),2003(1), 2004(5), 2005(5), 2006(11), 2007(6),2008(12) |
|  | H2(1) | 1994(1) |  | Neimeng | H1a(27) | 2005(5), 2006(7), 2007(10),2008(5) |
| Chongqing | H1a(24) | 2000(1);2001(2);2003(5);2004(5);2006(5);2007(6) |  | Ningxia | H1a(41) | 2004(2),2005(13), 2006(2), 2007(19),2008(5) |
|  | H1b(6) | 2000(4);2001(2) |  | Qinghai | H1a(9) | 2000(2),2004(2), 2005(5) |
| Fujian | H1a(4) | 2002(1);2007(3) |  | Shaanxi | H1a(19) | 2000(1),2001(4), 2003(2),2005(8), 2007(2),2008(2) |
| Gansu | H1a(13) | 2004(2);2006(7);2007(1);2008(3); |  |  | H1b(2) | 2000(2) |
| Guangdong | H1a(117) | 2001(3);2002(4);2004(1);2005(20);2006(39);2007(18);2008(32) |  | Shandong | H1a(175) | 1993(5),1997(1),1999(1),2000(3),2001(10), 2002(5), 2003(1), 2004(2), 2005(9), 2006(21), 2007(61), 2008(56) |
| Guangxi | H1a(19) | 2001(2);2006(1);2007(14);2008(2); |  |  | H1b(5) | 2000(2),2005(3) |
| Guizhou | H1a(15) | 2004(1);2007(12);2008(2); |  |  | A(2) | 1993(1),2007(1) |
|  | H1b(3) | 2004(3) |  | Shanghai | H1a(125) | 2000(1),2001(11), 2002(18), 2003(49),2004(2),2007(24),2008(20) |
| Hainan | H1a(20) | 2001(5);2003(7);2005(8) |  |  | H1b(6) | 2001(3),2002(2),2003(1) |
|  | H1b(2) | 1999(1);2003(1) |  | Shanxi | H1a(70) | 2000(2),2001(5), 2002(4), 2003(1),2004(12),2005(9),2006(27),2007(5),2008(5) |
| Hebei | H1a(68) | 1994(2),2003(1);2004(1);2005(19);2006(30);2007(8);2008(7) |  |  | H1b(6) | 2000(1),2001(2),2002(3) |
|  | H1b(3) | 2004(1),2005(2) |  | Sichuan | H1a(34) | 2002(1), 2003(9),2004(5),2005(5),2006(2),2007(12) |
| Heilongjiang | H1a(16) | 2002(1);2005(4);2006(7);2007(1);2008(3) |  |  | H1b(4) | 2003(1),2004(3) |
| Henan | H1a(182) | 1999(8),2000(11),2001(5), 2002(1),2003(4); 2006(1);2007(65), 2008(87) |  | Tianjin | H1a(57) | 2002(13), 2003(4),2004(5),2005(14),2006(16),2008(5) |
|  | H1b(9) | 1999(6);2000(1);2001(1);2002(1); |  |  | H1b(1) | 2002(1) |
|  | A(1) | 1999(1) |  | Xinjiang | H1a(8) | 2001(1), 2002(3),2003(2),2004(2) |
| Hubei | H1a(1) | 2008(1) |  |  | H1b(1) | 2004(1) |
| Hunan | H1a(37) | 1993(2),1994(1),1995(1), 2001(3), 2006(18), 2007(11), 2008(1) |  |  | A(1) | 2003(1) |
|  | H1b(5) | 1994(1),1995(3),2001(1) |  | Yunnan | H1a(21) | 2005(5), 2006(14),2007(1),2008(1) |
|  | A(1) | 1996(1) |  |  | H1b(2) | 2004(2) |
